# Improving bimanual interaction with a prosthesis using semi-autonomous control

**DOI:** 10.1186/s12984-019-0617-6

**Published:** 2019-11-14

**Authors:** Robin Volkmar, Strahinja Dosen, Jose Gonzalez-Vargas, Marcus Baum, Marko Markovic

**Affiliations:** 1 Department of Trauma Surgery, Orthopedics and Plastic Surgery, University Medical Center Göttingen, Von-Siebold-Str. 3, 37075 Göttingen, Germany; 2 Department of Health Science and Technology, Center for Sensory-Motor Interaction, Aalborg University, Aalborg, Denmark; 3 Department at Ottobock SE & Co, KGaA, 37115 Duderstadt, Germany; 4 Institute of Computer Science, University of Göttingen, Göttingen, Germany

**Keywords:** Myoelectric prosthesis, Myocontrol, Bimanual interactions, Inertial sensing, Sensor-fusion, Semi-autonomous control

## Abstract

**Background:**

The loss of a hand is a traumatic experience that substantially compromises an individual’s capability to interact with his environment. The myoelectric prostheses are state-of-the-art (SoA) functional replacements for the lost limbs. Their overall mechanical design and dexterity have improved over the last few decades, but the users have not been able to fully exploit these advances because of the lack of effective and intuitive control. Bimanual tasks are particularly challenging for an amputee since prosthesis control needs to be coordinated with the movement of the sound limb. So far, the bimanual activities have been often neglected by the prosthetic research community.

**Methods:**

We present a novel method to prosthesis control, which uses a semi-autonomous approach in order to simplify bimanual interactions. The approach supplements the commercial SoA two-channel myoelectric control with two additional sensors. Two inertial measurement units were attached to the prosthesis and the sound hand to detect the movement of both limbs. Once a bimanual interaction is detected, the system mimics the coordination strategies of able-bodied subjects to automatically adjust the prosthesis wrist rotation (pronation, supination) and grip type (lateral, palmar) to assist the sound hand during a bimanual task. The system has been evaluated in eight able-bodied subjects performing functional uni- and bi-manual tasks using the novel method and SoA two-channel myocontrol. The outcome measures were time to accomplish the task, semi-autonomous system misclassification rate, subjective rating of intuitiveness, and perceived workload (NASA TLX).

**Results:**

The results demonstrated that the novel control interface substantially outperformed the SoA myoelectric control. While using the semi-autonomous control the time to accomplish the task and the perceived workload decreased for 25 and 27%, respectively, while the subjects rated the system as more intuitive then SoA myocontrol.

**Conclusions:**

The novel system uses minimal additional hardware (two inertial sensors) and simple processing and it is therefore convenient for practical implementation. By using the proposed control scheme, the prosthesis assists the user’s sound hand in performing bimanual interactions while decreasing cognitive burden.

## Introduction

The human hands are essential tools for many activities of daily living (ADL). They are capable of dexterous yet reliable manipulation, firm grasping, and are instrumental for haptic exploration of the environment and social communication. Unfortunately, hand amputations are estimated to occur 18,496 times each year and an estimated total of 541,000 humans are affected by upper limb amputations in the United States alone [1]. Whereas cosmetic prostheses restore the appearance and overall body image, active prostheses can additionally restore different levels of functionality.

Modern active prostheses are typically controlled using electromyography (EMG) signals [2]. The surface EMG (sEMG) electrodes are placed on the skin surface to detect myoelectric signals generated by muscle fibers during contraction. The first myoelectric prostheses were introduced in the 60s and were simple single-DoF grippers [3]. They were controlled using two sEMG electrodes that were placed on a pair of antagonist muscles (e.g. wrist extensor and flexor muscles of the forearm) in order to directly control a prosthesis function (e.g., hand open/close). Therefore, only single degree of freedom (DoF) could be operated at the time, and a switching signal, such as muscle coactivation, had to be used to change the active DoF. As the mechatronic technology advanced, new solutions emerged and already in the 80s, there was active research performed on the concepts of under-actuated control mechanisms [4]. However, the translation of research efforts to commercial realm lost its initial momentum since it was recognized that main problems of upper limb prosthetic systems lie in the limitations of available man-machine interfaces [5, 6]. Namely, the two EMG-channel control although relatively robust, turned out to be slow and tedious when applied to multi-DoF prostheses [7]. Therefore, development of prosthetic hands slowed down and stayed behind the modern robotic technology. In the last decade, prosthetic hands with multi-articulated fingers have been commercially introduced [8]. However, due to their high mechanical complexity and poor, under-developed control interface the overall robustness suffered, thus rendering under-actuated prostheses popular to this day [9]. Moreover, the new prostheses focused on replacing the finger function whereas joints such as wrist received only limited attention and continued to provide rather limited, single-axis functionality [8]. Only with the recent commercial introduction of the myoelectric machine learning interfaces [10] a new impulse is given for further development and improvement of the prosthetic hands. Machine learning methods can be used to improve prostheses control as they rely on recording and classifying the activity from multiple muscles to directly activate desired DoFs in a coordinated manner [11]. However, their implementation remains challenging due to decreased robustness and increased learning curve [8, 12] in comparison to the standard two-channel control. To increase robustness, myoelectric control has been enhanced by adding additional sensors to the prosthesis [13]. In several studies, inertial measurement units (IMU) were used as extra inputs into a classifier [14] or to select an appropriate classifier based on the limb position [15, 16]. In [17], an IMU has been employed to control wrist rotation (pronation, supination) through upper arm movements, while grasping has been operated using the classic myoelectric interface. A combination of sensors embedded into a prosthesis (force and angle) and an IMU has been applied in [18] to estimate the state of the prosthesis and adapt myoelectric control accordingly. Additional sensors can be also used to implement automatic control of prosthesis functions, thereby decreasing cognitive burden from the user. For example, in [19–21] a myoelectric prosthesis was equipped with computer vision enabling automatic grasp type and size selection. Computer vision has been combined with an IMU in [22] to control wrist rotation in addition to grasping. In that system, the IMU has been used to measure user movements, as he/she orients the prosthesis using proximal joints (shoulder and elbow). The automatic controller was thus able to react online to user actions and reconfigure the grasp parameters based on the side from which he/she approaches the object. These studies have demonstrated that semi-autonomous control is indeed a promising approach, which can simplify and improve prosthesis control. In [22], the semi-autonomous system significantly outperformed the classic manual control when operating three degrees of freedom and it also reduced compensatory motions.

Most studies in prosthesis control, either manual or semi-autonomous, focus on the unilateral tasks, where a prosthesis is used to grasp and manipulate an object, without participation of the sound hand. However, many important activities of daily life are normally employed bimanually, for example, picking up a larger object or transferring an object from hand to hand. Able-bodied subjects accomplish these tasks routinely by exploiting the dexterity available in both limbs. Both hands actively collaborate while performing the task. The amputees, on the other side, chose to compensate the bodily deficit by over-employing the unaffected side [23, 24]. In bimanual activities, they often use the prosthesis sub-optimally, e.g., as a passive support to the sound limb. This is due to difficulties in controlling the prosthesis, where switching between the functions and adjusting the degrees of freedom would substantially increase the time and effort needed to perform a bimanual task. In [25], bimanual and unimanual tasks were performed by able-bodied subjects using prostheses mounted bilaterally. Bimanual tasks almost doubled the execution times and significantly reduced the success rates. Consequently, an active prosthesis could significantly enhance the quality of amputee’s life if it would enable him to perform bimanual interactions with greater ease.

**Fig. 1:**
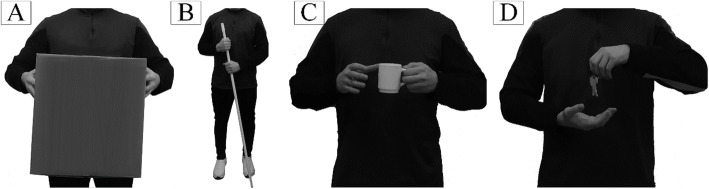
Examples of common bimanual interactions in which both hands participate: **a**) carrying a bulky object, **b**) holding onto something or using a tool with both hands, and **c** and **d**) transferring an object from one hand to another

Importantly, when performing bimanual interactions with an object, in many cases the two hands move in a coordinated and stereotyped manner (Fig. 1). For example, to lift a heavy box, the hands move symmetrically (Fig. 1a), while to transfer an object from hand to hand, the hands mirror each other (anti-symmetric motion, Fig. 1d). This mechanism is crucial for natural interaction and fluidity of motion [26, 27].

The fact that the hands move in consistent patterns during bimanual tasks makes this an ideal context for implementing semi-automatic assistance to prosthesis control. In this study, we present a novel system for semi-autonomous bimanual prosthesis control (BPC). The system to automatically adjusts prosthesis wrist rotation (pronation, supination) and grasp type (lateral, palmar) by classifying hand interactions either as unilateral, synchronous-bimanual or asynchronous-bimanual. The BPC was evaluated experimentally using functionally relevant tasks, and the results demonstrate that it substantially enhances the execution performance of bimanual interactions while using a myoelectric prosthesis.

## Material and methods

### System components

**Fig. 2:**
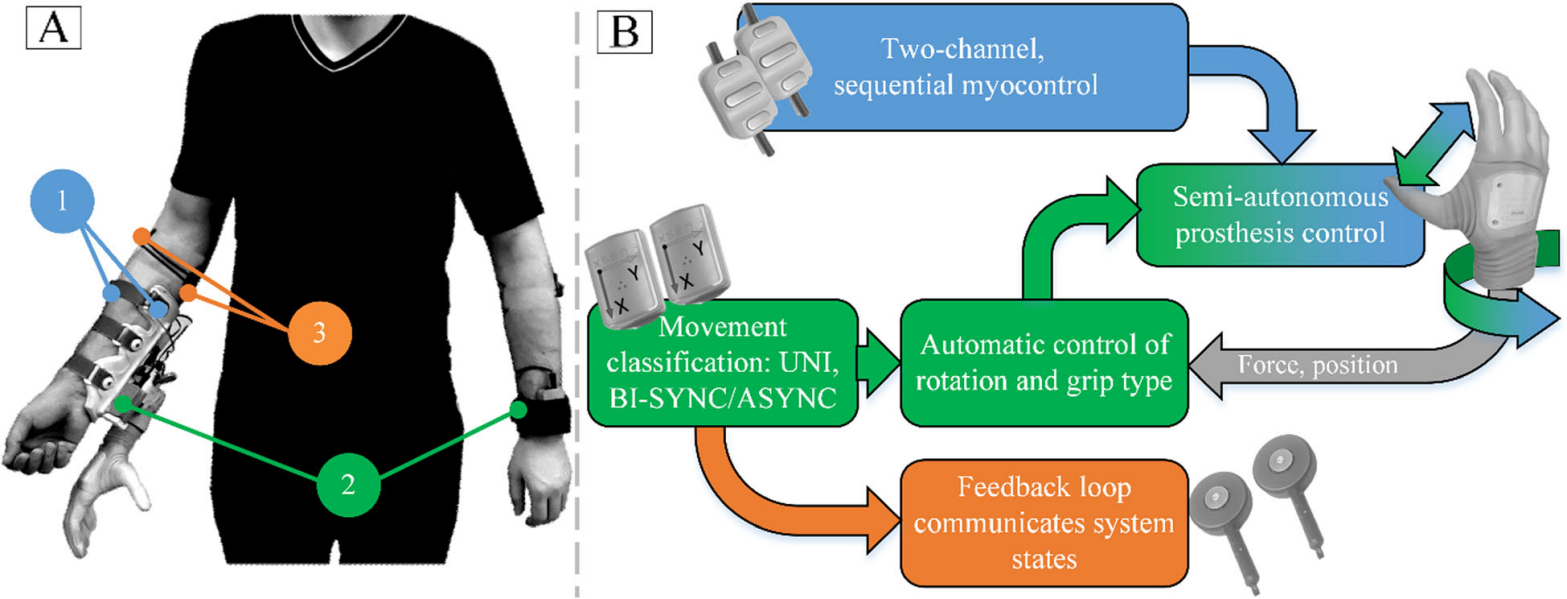
Overview of the semi-autonomous bimanual prosthesis control system (BPC). **a**) System components; **b**) Processing pipeline; Annotations: (1) sEMG electrodes (2) IMUs (3) Vibrotactors

The BPC prototype consists of the following components (Fig. 2a): (1) two dry sEMG electrodes with embedded low-pass filters that output the linear envelope of the EMG signal (OB 13E200, OttoBock GmbH, Germany). The electrodes are placed on the skin above wrist flexor and extensor muscles; (2) two inertial measurement units - IMUs (Xsens Technologies B.V., Enschede, NL) positioned on the prosthetic hand and the contralateral hand to measure the 3-axial acceleration and orientation; and (3) two vibrotactors (C2-Tactors, Engineering Acoustics, Inc., FL, USA) placed on the skin proximal to the elbow on medial and lateral side of the forearm. The system was evaluated using the commercially available Michelangelo hand prosthesis (OttoBock GmbH, Germany) with a wrist rotation unit and embedded position encoders and force sensors. The prosthesis has three degrees of freedom (DoFs): two grip types (palmar and lateral) and a wrist rotation. All components were connected to a standard desktop PC (8GB RAM, i5 processor, Win 7 OS) running custom software written in MATLAB 2015b for data acquisition and real-time control. The control loop executed at 50 Hz and all sensors communicated at 100 Hz refresh rate. The Bluetooth delay of the prosthesis and EMG acquisition system was around 80 ms [28] whereas the delay of the Xsens communication is negligible since it uses proprietary wireless interface [29].

Michelangelo prosthesis is an advanced, commercially available prosthesis that can perform two grip types with relatively high force (70–100 N) and actively rotate the wrist [30]. Moreover, Michelangelo hand has embedded force and position encoders, which allow closed-loop position control. The hand itself operates using under-actuated mechanism with two motors. One motor acts as a main drive and is responsible for closing and opening all fingers simultaneously. The other motor is a dedicated thumb drive, which can change the thumb position and therefore switch the current grip type between lateral and palmar. In palmar preshape, the thumb positions below the palm in order to form opposition allowing thereby a high-power grip around an object. In lateral preshape, the thumb moves to the plane of the palm and closes against the lateral aspect of the index finger forming a grip similar to that of holding a key or a credit card. Furthermore, by fully opening the prosthesis in lateral preshape the fingers reach a position that reassembles that of a naturally relaxed human hand and this can be used to hold objects like trays or plates [31]. Importantly, the prosthesis firmware does not allow for low-level (individual) control of the embedded motors, rather it implements internal control loop that takes care that the motors act synergistically according to the user-selected grip type.

**Fig. 3:**
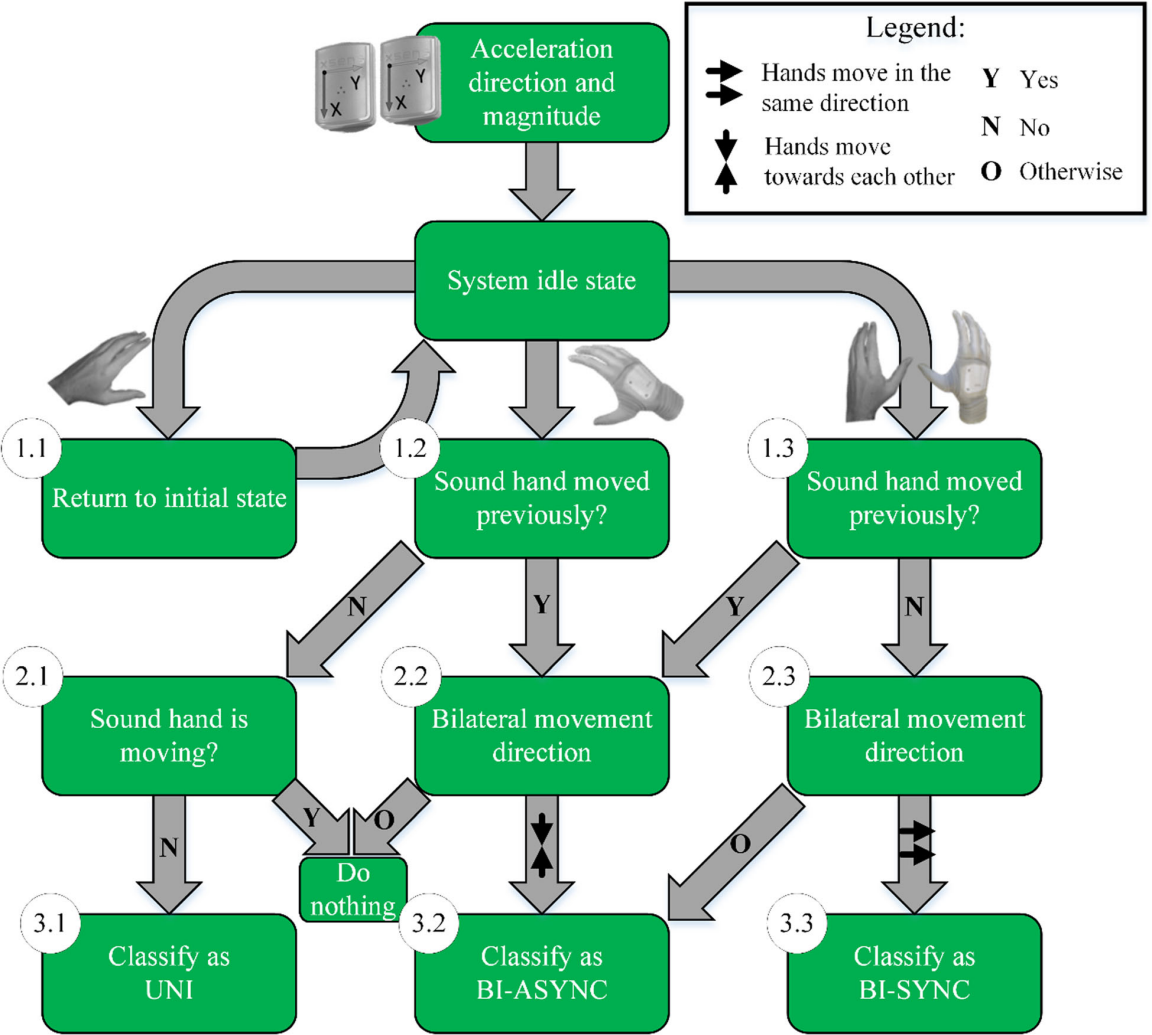
Movement classification. The magnitude and direction of acceleration is processed by a set of IF-THEN rules to classify the movement as unilateral (UNI), bimanual asynchronous (BI-ASYNC) or bimanual synchronous. (BI-SYNC).

The BPC system consists of 1) an autonomous controller (green, Fig. 2b), which determines the prosthesis orientation and grip-type based on sensor data from Michelangelo hand prosthesis (force and position encoders) and IMU sensors that provide 3D acceleration and internally estimated 3D orientation (firmware implemented Kalman filtering) for both forearms in global coordinate system [29], 2) myoelectric interface for manual control (blue, Fig. 2b), and 3) tactile feedback communicating the state of the system to the subject (orange, Fig. 2b). The autonomous controller is active only when the prosthesis is not actively operated by myocontrol (Fig. 2b). The myocontrol has the priority over the autonomous controller, which means that the user can always override the autonomous decisions using myocontrol; hence, the overall control scheme is semi-autonomous. Finally, the control loop is closed by means of vibrotactile feedback that communicates the state of the autonomous system back to the user. This includes the estimated type of interaction and the system-ready state (see section 2.2.2).

### System operation

The overall control loop is shown in Fig. 2b. In the idle state, the system waits for the subject to start the movement. When the movement is detected, the system classifies the movement into three interaction classes: unimanual prosthesis movement (UNI), bimanual synchronous movement (BI-SYNC) and bimanual asynchronous movement (BI-ASYNC). In BI-SYNC, both hands are moved approximately synchronously to simultaneously grasp and manipulate an object (e.g., as depicted in Fig. 1a and b). In BI-ASYNC, the sound hand grasps an object and transfers it to the prosthesis (e.g., Fig. 1c and d). The outcome of the movement classification determines the response of the autonomous control system. Each movement type activates a specific automatic control strategy coordinating the movement of the prosthesis to that of the contralateral hand. The automatic controller continuously adjusts the orientation of the prosthesis and its grasp type. When ready for grasping, the subject closes the hand using myoelectric control, and when the contact is detected, the automatic control is deactivated. The subject can then manipulate the object using myoelectric control and after the object is released, the system transits back to the initial state.

#### Myoelectric control

The BPC utilizes direct myocontrol with sequential switching between the DoFs, which is a standard solution in commercial systems [32]. The subject can control one DoF at a time, in a proportional manner. To switch between the hand and wrist control the user needs to generate a trigger signal by strongly activating and quickly releasing the wrist extensor muscles (thereby producing a “myoelectric” impulse). In order to switch between the different handgrip types (palmar, lateral) the user needs to generate the same kind of trigger signal using the wrist flexor muscles. Therefore, the user is able to quickly switch between two primary prosthesis functions (wrist rotation and hand open/close) using a single trigger. This myocontrol scheme is commercially available and used by some amputees who use multi-functional hand prostheses [33].

#### Movement detection and classification

The magnitude of acceleration and its direction are used to detect the motion of the prosthesis and the sound hand, and to distinguish between the three types of movement (Fig. 3). The acceleration magnitude is compared to heuristically determined thresholds, which are adjusted for each subject individually. While in the idle state, the system continuously monitors the acceleration to detect when the hand and the prosthesis start moving. If only the sound hand moves, the algorithm makes no decision, buffers the hand movement for 500 ms and waits for further input (block 1.1). This was implemented in order to recognize those interactions that are bimanual but do not start with simultaneous movement of both limbs. For example, the object transfer from the sound to the prosthetic hand is commonly performed by first moving the sound hand to pick up an object and then the prosthetic hand to receive the object from the sound hand. In order to distinguish this interaction from a simple unilateral movement, the system continuously tracks the sound hand over 500 ms and determines if it has moved. Put differently, this approach establishes a maximal time window in which the movement of the two hands needs to occur in order to be considered as a potential bimanual interaction. If the prosthesis moves, while the sound hand is not moving (i.e., no movement detected in last 500 ms), the algorithm checks if the hand has previously moved using the hand movement buffer (block 1.2). If this is not the case, and the hand remains static, (block 2.1), the movement is classified as unilateral (UNI) with the prosthesis (block 3.1). However, if the sound hand has moved in the last 500 ms, and the prosthesis and hand are now moving towards each other (block 2.2), the movement is classified as BI-ASYNC (block 3.2). Finally, if the system is in the idle state and detects that both the sound hand and prosthesis move at the same time and the hand has moved before (block 1.3), the moving directions are checked. If the hand and prosthesis move towards each other, the movement is classified as BI-ASYNC case (block 2.2). If both the sound hand and prosthesis start moving but the hand has not moved before, the movement can be classified as either BI-SYNC or BI-ASYNC, depending on the direction of the movement (block 2.3).

#### Automatic control

**Fig. 4:**
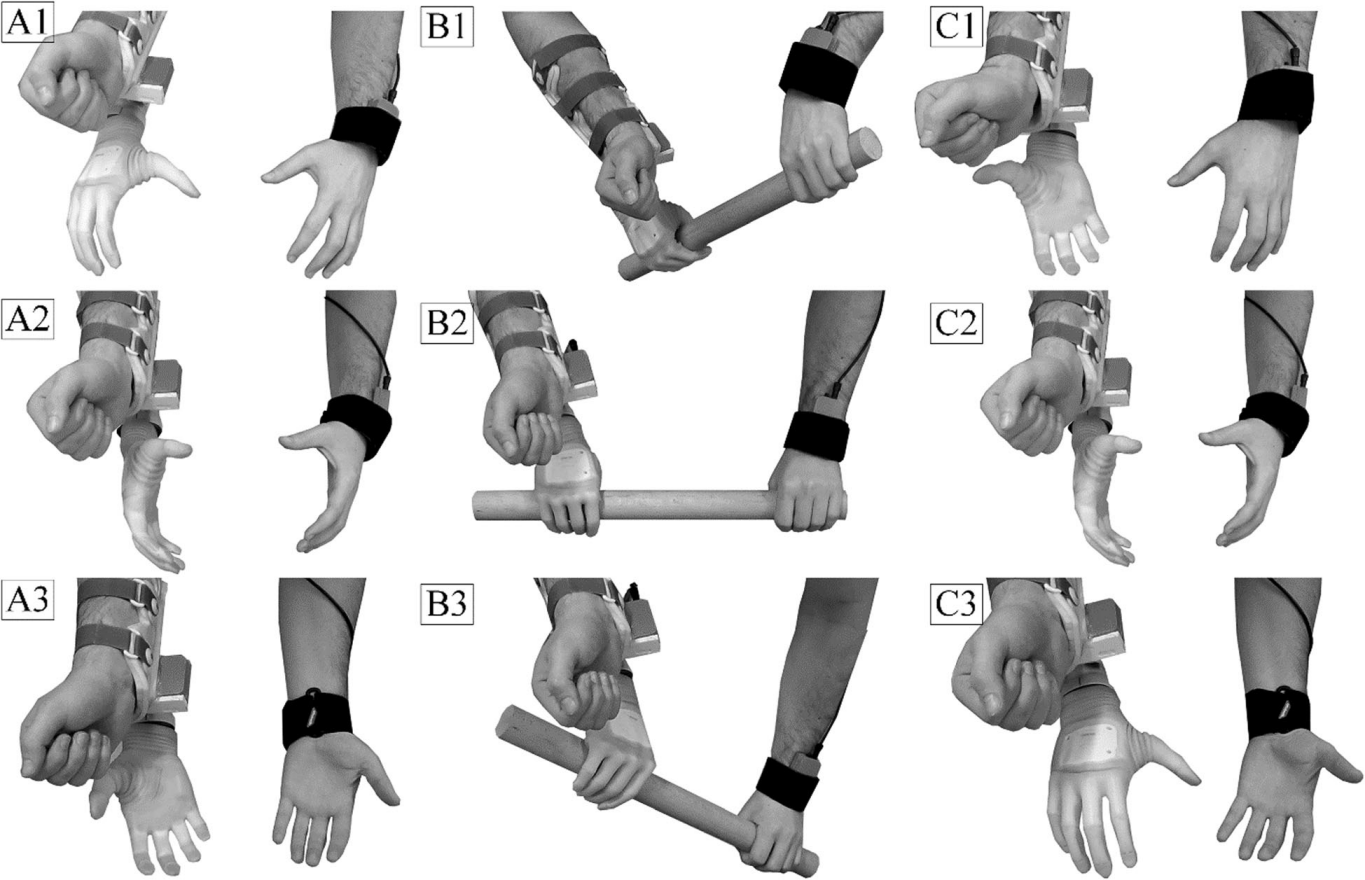
Prosthesis behavior is automatically regulated during the bimanual interactions. **a**1–3) In BI-SYNC mode, the prosthesis automatically rotates congruently to the sound limb and adjusts its grip type, so that the object can be grasped with both hands. **b**) In BI-SYNC mode, after the object has been grasped, the prosthesis rotation continues to be atomatically adjusted to facilitate bimanual manipulation. **c**) In BI-ASYNC mode, the prosthesis automatically matches the rotation of the sound hand so that the palms of both hands always point towards each other in order to facilitate successful object transfer. After the object has been grasped, the control is switched to manual

The autonomous controller is activated after the movement is classified and it operates differently in each of the movement classes (Fig. 4). The force sensor embedded in the prosthesis is used to detect object grasp and release. If the movement is classified as UNI, the autonomous controller assumes that the subject will use only the prosthesis to start and finish the interaction with the object. In this scenario, the prosthesis is operated manually. However, the wrist pronation/supination can be adjusted either via myocontrol or by changing the shoulder inclination angle (adduction/abduction) measured by the IMUs as described in [17]. The IMU control of the wrist was implemented in order to exploit the additional sensor available in BPC.

During BI-ASYNC movements, the autonomous controller expects that the user will transfer an object from the sound hand to the prosthesis. In order to facilitate this interaction, it automatically adjusts the prosthesis rotation to match the rotation of the sound limb so that the palms always face opposite directions (Fig. 4c1-3). The prosthesis stops following the sound limb as soon as the object transfer is complete. Therefore, after the object is grasped by the prosthesis, the automatic control is deactivated and the prosthesis is controlled manually.

During BI-SYNC movements, the autonomous controller assumes that the user would grasp an object using both hands and therefore, it automatically adjusts the prosthesis rotation to match the rotation of the sound limb so that the wrists move symmetrically (Fig. 4a1, 3). Furthermore, the controller also automatically switches between the palmar and lateral grip type, depending on the orientation of the sound hand. If the hand is rotated downwards or to the side, the prosthesis assumes palmar preshape since it is expected that the user will grasp something with both hands and for this, he needs to achieve a secure, stable grip (Fig. 4a1-2). However, if the sound hand is rotated upwards, then the prosthesis automatically changes the preshape to the neutral (lateral fully open) since it is expected that the user could carry an object without necessarily forming a grip around it (e.g., a tray, Fig. 4A3). Additionally, since closing in lateral preshape moves the thumb slightly towards the inner side of the palm, the user could also choose to slip the object between the thumb and the palm and then slightly close the prosthesis in order to form a secure grip around it (e.g., a tray, Fig. 4A3). Finally, in order to facilitate manipulation in the BI-SYNC mode, the system continues to automatically regulate the rotation after the object is grasped by rotating the prosthesis wrist asymmetrically to the sound hand (Fig. 4b1-3).

#### Feedback

**Fig. 5:**
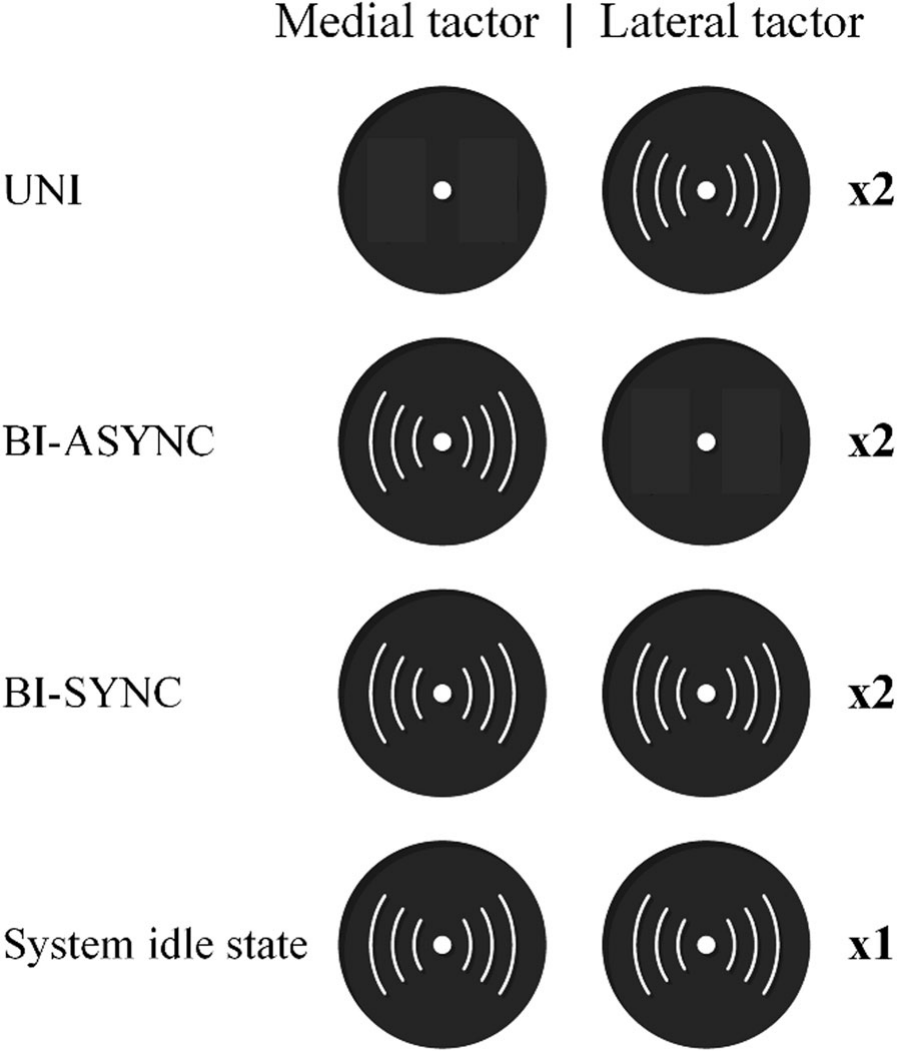
Feedback coding scheme used to indicate the system’s current state. Depending on the state, the tactors can vibrate either once (× 1) or twice (× 2). UNI, BY-ASYNC and BI-SYNC denote that unilateral, bilateral asynchronous and bilateral synchronous movements were detected by the system, and that the corresponding automatic control specific to the detected class was activated

The vibration feedback communicates the following events to the user: 1) the system has recognized UNI interaction, 2) the system has recognized BI-ASYNC or 3) the BI-SYNC interaction and 4) the system is in the idle state – the system has detected that an interaction has ended (object released) and is ready to detect another one. These events are coded using single or double vibration burst as depicted in Fig. 5. The lateral tactor represents the prosthesis and the medial the sound limb. Therefore, the double vibration burst of the lateral tactor indicates that the prosthesis movement has been recognized (UNI), whereas the double burst of the medial tactor indicates the movement of the sound limb in the context of BI-ASYNC. Finally, both tactors are activated with a double burst in order to indicate that BY-SYNC has been recognized or with a single burst to indicate that the system is ready to recognize the next motion (back to idle state).

### Experimental evaluation

The BPC was compared to the conventional, two-channel myocontrol (commercial SoA). Therefore, the myocontrol algorithm used for comparison to the BPC was identical to the one implemented within the BPC system. The experimental evaluation addressed four different interaction scenarios: unimanual interactions using the sound hand, unimanual interactions using the prosthesis and two variations of bimanual interactions (synchronous and asynchronous).

The study was approved by the Ethics committee of the University of Göttingen (approval number 22/04/2016). All experiments were conducted in accordance with the declaration of Helsinki, and all subjects signed a written informed consent prior to participation in the experiments. Eight able-bodied subjects were recruited for the study (age: 18–39; 3 females, 5 males). Two out of eight subjects were naïve to myoelectric prosthesis control. Each subject performed the experiment in two conditions: once using the SoA myoelectric control and once using the BPC.

#### Experimental task

**Fig. 6:**
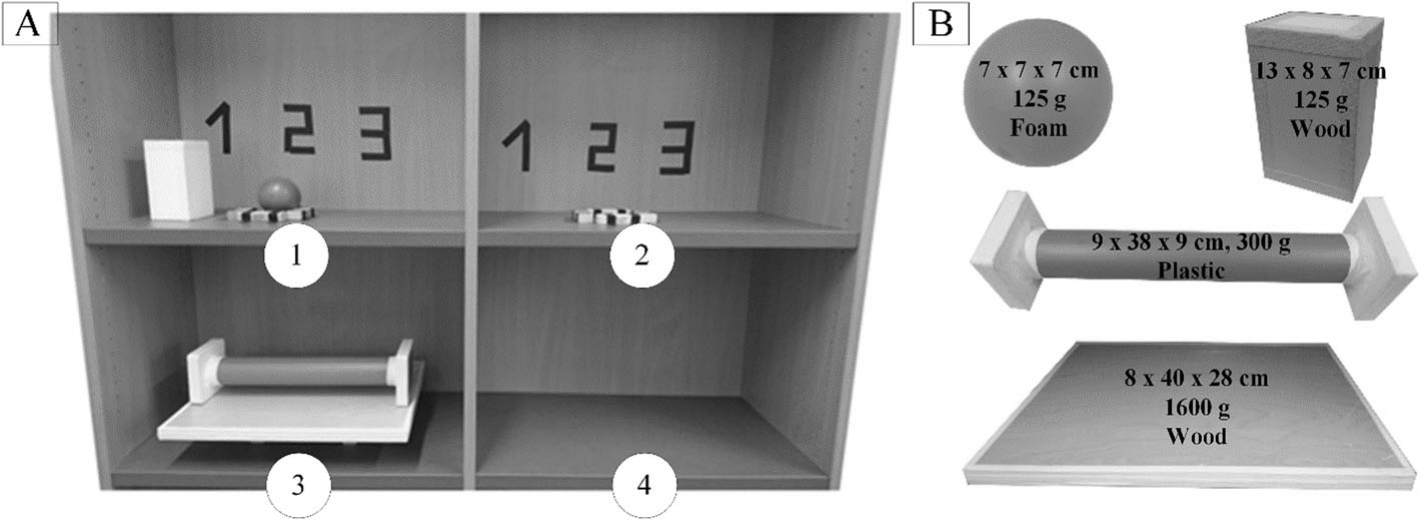
Overview of the experimental task (**a**) and objects used in the experiment (**b**). The dimensions of objects are presented as height x width x depth

The subject performed the experimental task standing in front of a tall closet with four equidistantly placed shelves (Fig. 6a). Subject’s distance to the shelves ranged from 60 cm to 90 cm depending on the subject height. He/she faced the vertical intersection between the upper two shelves. The shelves were adjusted according to the subject’s height so that the lower shelves were at his/her hip-level and the upper shelves were at the midpoint between his/her chest and shoulder-level.

The overall task was to grasp and move objects, one by one, from one shelf to another. Four objects were used in the experiment (Fig. 6b): a box, a ball, a tray and a pipe. The upper shelves (annotated one and two) contained three numbered positions indicating the start and end position of each individual object (i.e., an object placed on position 1 to 3 should be moved to the same position on the opposite shelf).

One experimental trial consisted of seven interactions (Table 1) with four objects. Each interaction comprised the following steps: 1) reach for an object; 2) grasp the object; 3) manipulate it; 4) move and release the object on the contralateral shelf. The object, initial and final position, end-effector, grasp location, and type of manipulation for the seven possible interactions are specified in Table 1. For example, in interaction 3, the user grasped a box that lies at position 1 on the shelf 1 (Fig. 6 A1), with a sound hand and transferred it to the prosthetic hand (BI-ASYNC interaction). Then he/she needed to reach for the shelf 2 (with a prosthesis) and release the object at the position 1 (Fig 6 A2). The interaction order presented in the Table 1 (from interaction 1 to 7) is only one out of three possible variations used in the experimental protocol, as explained in the next section. The other two variations implemented the following order of interactions: 3, 1, 2, 4, 7, 5, 6 and 2, 3, 1, 4, 6, 7, 5.

**Table 1:** The experimental trial comprised of seven object interaction

Interaction number& type	Target object	Object start location	Reach the object with	Grasp the object from	Manipulate the object by	Object target location
1; BI-SYNC	Pipe (horizontal)	Shelf 3;	Synchronously bilaterally	Above; palmar grip	Rotating it to vertical orientation	Shelf 2; Position 3
2; BI-ASYNC	Ball	Shelf 1; Position 2	Sound hand unilaterally	Above; palmar grip	Rotating it 180° and handing it over to the prosthesis	Shelf 2; Position 2
3; BI-ASYNC	Box	Shelf 1; Position 1	Sound hand unilaterally	Side; palmar grip	Handing it over to the prosthesis	Shelf 2; Position 1
4; BI-SYNC	Tray	Shelf 3;	Synchronously bilaterally	Below; lateral grip	–	Shelf 4;
5; BI-SYNC	Pipe (vertical)	Shelf 2; Position 3	Synchronously bilaterally	Side; palmar grip	–	Shelf 1; Position 3
6; UNI (hand)	Ball	Shelf 2; Position 2	Sound hand unilaterally	Above; palmar grip	–	Shelf 1; Position 2
7; UNI (prosthesis)	Box	Shelf 2; Position 1	Prosthesis unilaterally	Side; palmar grip	Rotating it to horizontal orientation	Shelf 1; Position 1

#### Experimental protocol

The two experimental conditions (SoA myocontrol, BPC) were evaluated on separate days (sessions) in a randomized order. A session was divided in three phases: 1) introduction and system setup, 2) running the experiment and 3) filling out the questionnaires. Upon performing both experimental conditions, we performed an additional pilot test on three study participants in which they executed the experimental tasks without the prosthesis, using their own hands. This test was performed to obtain a reference performance for task execution in able-bodied subjects.

A session started with a general introduction to the prosthesis operation and overall experimental task. Since both experimental conditions rely on myoelectric control, the myocontrol scheme was explained at the beginning. In the BPC condition, the semi-autonomous control and feedback were explained in the context of the two types of bilateral interactions. The objects were presented and experimental task explained (Table 1). Next, the myocontrol was calibrated by measuring the maximal voluntary contraction (MVC) and setting the activation and trigger thresholds to 70 and 40% MVC, respectively. The IMUs in the BPC system were calibrated with elbows flexed at 90° and palms pointing towards each other. The thresholds for movement detection were set heuristically by asking the subject to perform five unimanual and bimanual (asynchronous and synchronous) interactions from Table I. The thresholds were then set manually by the experimenter so that other movements (e.g. moving the torso) did not trigger the system, while the hand and prosthesis movements did. The subject practiced using the selected control system by executing several pilot trials and the myocontrol/movement detection thresholds were then fine-tuned. In addition to this, the subjects were trained to interpret the vibrotactile feedback in the BPC condition. The tactors were activated and the subject was asked to interpret the meaning of the vibration according to the Fig. 5. This has been repeated until the subject achieved 100% recognition rate in 10 consecutive trials.

Before starting the experiments, the subjects were instructed that the overall goal of each trial is to perform the object interactions (Table 1) as fast as possible and without making any errors (e.g., object drops). The subjects trained the experimental tasks until they could perform two consecutive trials without errors. As previously stated, one trial consisted of seven correctly executed interactions. If an object was dropped, the trial started from the beginning; however, the drop was logged in order to calculate the total number of drops and thus quantify the amount of gross errors. The experiment included three sequences of two blocks, where each block included five trials. All the trials performed in a single sequence used one of the three variations in the order of interactions. The mapping between sequences and variations were pseudorandomized across subjects. In the first block of a sequence, the objects were placed in the left half of the closet (shelves 1 and 3) and moved to the right half (shelves 2 and 4), whereas in the second block the objects were moved in the opposite direction (from the right- to the left-half). Overall, this accounted to 30 trials (five trials × two blocks × three sequences), i.e. 210 object interactions (30 trials × seven interactions) per control condition (BPC, SoA).

At the end of each session, the subject answered the NASA task load index (TLX) questionnaire [34], which measures the perceived workload. Afterwards, he/she filled out a brief questionnaire, including four items on a visual-analogue scale (100 points, 5 points resolution) that addressed the usefulness of the vibro-tactile feedback in the BPC system as well as the overall intuitiveness of both SoA and BPC control systems.

#### Outcome measures and data analysis

In order to avoid influence of the learning curve on the performance metrics, only the last three trials of each block were used for data analysis, hence 18 trials per each control condition (1008 trials in total for eight subjects). The data was analysed by calculating the average performance of a subject across all included trials. Since the data did not pass the normality test (Lilliefors test), the Wilcoxon signed-rank test was applied to assess significant differences between the conditions. The statistical significance threshold was set to *p* < 0.05. The outcome of each performance metric was presented as the median (interquartile range).

The following outcome measures were used to compare the two control systems: 1) the average time the subject needed to complete an experimental trial; 2) the number of dropped objects; 3) perceived workload; 4) perceived intuitiveness of the control system. In addition, the accuracy of movement classification was evaluated for the BPC system. The experimenter monitored the task execution during the experiment and noted each occurrence when the BPC made a classification error, i.e., the system decision did not match the movement class indicated in Table 1. The misclassification rate was calculated as the ratio between the misclassified and the total number of movements for each of the three possible classification outcomes (UNI, BI-SYNC, BI-ASYNC).

## Results

**Fig. 7:**
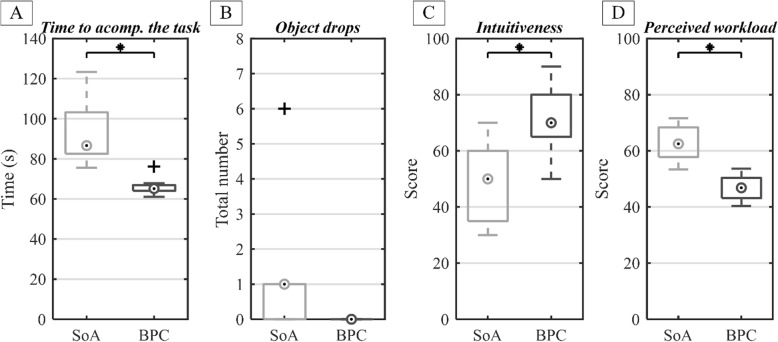
The summary performance of the two control schemes, namely, novel bimanual prosthesis control (BPC) versus commercial state of the art myoelectric control (SoA): the time to accomplish the trial (**a**), total number of object drops (**b**), system intuitiveness (**c**) and perceived task workload (**d**). The median is annotated by a circle and the interqurtiale range (IQR) is represented by the box. The whiskers are the minimum and maximum values, and the crosses are the outliers. The star above the boxplots indicates statistically significant difference (*p* < 0.05)

The subjects were significantly faster when performing the experimental task (Fig. 7a) with the BPC compared to the SoA myocontrol. The time to accomplish the experimental trial was 65(3) s vs. 87(21) s for BPC and SoA, respectively. Subjects did not drop any of the objects in the BPC condition (Fig. 7b); however, this was not significantly different from the SoA condition, where the median number of dropped objects was one. The subjects rated BPC substantially more intuitive (Fig. 7c) to use. The subjective rating was 70(15) vs. 50(25) for BPC and SoA, respectively. Likewise, the overall task workload was significantly lower (Fig. 7d) in BPC than in the SoA condition, i.e., the NASA TLX workload index was 47(7) vs. 63(11) for BPC and SoA, respectively.

**Fig. 8:**
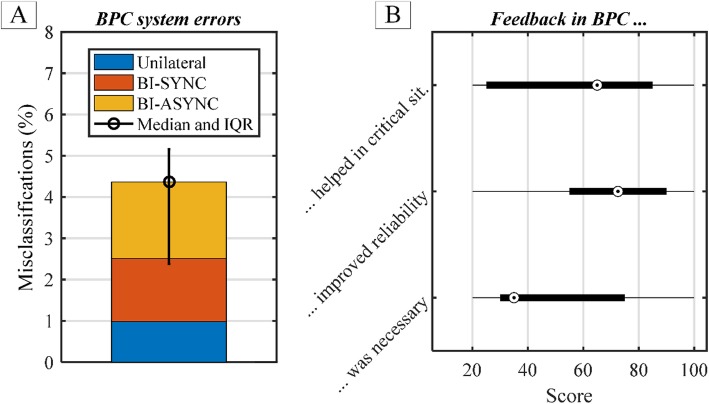
Movement misclassification rate (**a**) and feedback evaluation in BPC condition (**b**). The circles represent the median and black lines the interqurtiale range (IQR). The total missclasification error is calculated as the sum of individual errors for UNI, BI-ASYNC and BI-SYNC movements

The BPC system correctly classified the limb movements in 95% of the 1008 interactions. The total misclassification rate (Fig. 8a), averaged across subjects, was 4.4(2.5)%. More specifically, misclassifications of UNI movements accounted for 23%, BI-SYNC for 35% and BI-ASYNC for 42% of the total misclassifications. Observed individually, the BPC system achieved the misclassification rate below 5% for majority of the subjects (seven out of eight). Only in one subject, the misclassification error rate was around 8%. The subjects deemed the feedback as helpful but not necessary (score 35(45)) for the overall system operation (Fig. 8b).

Finally, the three subjects, who used their own hands to accomplish the task, significantly outperformed both SoA and BPC condition. The median trial time in this condition was 17(0.3) s which was five and four time faster than in SoA and BPC condition, respectively.

## Discussion

We have developed a semi-autonomous system for bimanual prosthesis control (BPC) and compared it to the commercial SoA myocontrol in eight able-bodied subjects performing a variety of unilateral and bilateral object interactions. The results demonstrated that BPC substantially improved the interactions between the sound hand and a prosthesis by significantly decreasing the time to accomplish the task and perceived workload. In addition, the use of BPC improved the performance consistency across subjects, as evidenced by the lower variability of the time to accomplish the task (Fig. 7A). It is known that there is a substantial difference in myoelectric control skills among different subjects [12, 35]. In the present experiment, the subjects were additionally challenged by the fact that they needed to operate the prosthesis in coordination with the sound hand. This has resulted in substantial variability in performance with myoelectric control, where the time to accomplish the task ranged from 75 to 123 s. However, with BPC, most of the control and coordination has been taken over by the automatic system, activated by movement detection and classification. This has decreased the cognitive burden from the subjects as well as equalized the performance, since individual skills were not anymore relevant for system operation. Consequently, all subjects but one outlier could accomplish the task in almost the same time (between 61 and 67 s), which corresponds to eightfold decrease in variability with respect to myocontrol. In the case of UNI, the subjects could control the wrist either using myoelectric control or an IMU through shoulder movements. We have noticed that the latter possibility has been used by most of the subjects, and that this has improved the performance in UNI mode. However, only a single interaction was of this type (Table 1, column 7), and therefore, the contribution of the IMU wrist control to the time to accomplish the trial was overall modest. Nevertheless, we have decided to implement this approach since it further demonstrates the advantage of prosthesis control systems that rely on multimodal sensing. Importantly, experimental tests showed that BPC was not biased towards bilateral interactions, since only few of unilateral movements were wrongly classified as bilateral (Fig. 8A). More specifically, only 23% of all errors were attributed to misclassification of UNI as BI. Furthermore, although we did not directly measure it, we have not observed that the users intervened and corrected decisions of the BPC system, except for the very few erroneous misclassification cases. Finally, the subjects dropped objects very rarely during the experiment. This is due to the fact that most tasks were performed using both hands, thereby providing safety and stability (especially by the sound hand).

The subjects also experienced the system as more intuitive than the SoA myocontrol. This is likely because the BPC allowed the subjects to perform bimanual tasks smoothly, mimicking thereby natural movements. When using myoelectric control, the subjects were forced to make breaks to activate the prosthesis functions as required by the task (wrist rotation and grasp type). In BPC, the prosthesis parameters were configured automatically, and the subjects only needed to move and position the prosthesis. Importantly, the parameters were adjusted using natural strategies that characterize the coordination of sound hands during bimanual tasks of able-bodied subjects. Therefore, the prosthetic hand behaved as intuitively expected by the subjects.

The simple method that has been used to detect and classify unimanual and bimanual movements showed to be very effective, resulting in a low rate of misclassifications while being tested in a range of functional tasks. This is important as the algorithm can be easily implemented in an embedded prosthesis controller. The method has to be calibrated individually for each subject, but this calibration was simple and fast (less than 20 min). Importantly, the system provided the subjects with vibrotactile feedback communicating the results of movement detection and classification. Therefore, the subjects were explicitly notified in case of misclassification, so that they could take over the control (semi-autonomous system) and prevent the unexpected behaviour of the hand. The subjects found this feedback useful but not necessarily required for the system operation. The latter is likely due to the high reliability of classification in the first place. If the classification has been less reliable, the feedback would be likely experienced as more critical by the subjects. Overall, the subjective estimations of the feedback utility were highly variable, indicating that the approach to feedback was subject specific.

Although the presented experimental tasks could be considered simple from the able-bodied perspective their relevance is expressed in the very fact that they are still quite difficult to perform using classical (myoelectric) control schemes. Namely, using their own hands the subjects performed the task five and four times faster than in SoA and BPC condition, respectively. Therefore, similar to observations made in [9, 36] the experimental tests in this study demonstrate how big the gap between natural and prosthetic hand function truly is, especially while performing bimanual tasks. This points out not only the necessity of further improving the interaction capacity between sound and the affected limb but also at designing novel tests that are able to quantify this [37]. And indeed, most of the tests that are used in the prosthetic literature (e.g., Box and blocks, Clothespin test and SHAP) comprise unimanual activities with limited range of motion [38]. Therefore, for the experimental assessment in the present study, we have selected a set of bimanual tasks that are representative of a large class of daily life activities (e.g., picking up objects with both hands, object transfer from hand to hand, supporting a tray). In conclusion, in the context of prosthesis evaluation, the experimental tests presented here can be considered not only unique and novel but also highly relevant.

The BPC uses only two IMU sensors but it is not self-contained, as one of the sensors needs to be placed on the sound hand. However, this is not an important drawback because wearable sensing is becoming more and more pervasive, and the contralateral IMU could be exchanged with a smart watch, fitness brace or a similar gadget. The system presented in this study supports some important types of bimanual tasks, but not all of them, and it is yet to be developed into a solution that can be used robustly in a clinical environment. For example, in some bimanual activities the spatial coupling between the hands will be neither symmetric nor anti-symmetric, as when grasping a jar by the side and a lid from the top in order to open/close the jar. Therefore, the BPC needs to be expanded with additional strategies and scenarios in order to support a greater variety of activities and movements (e.g., initiating reach and grasp while walking towards a table). To this aim, a future system could be combined with machine learning [39] or with some of the recently presented systems for unimanual prosthesis control that are based on computer vision [19–21]. This would lead to an advanced system for the control of dexterous prostheses that would support the user while performing both unimanual and bimanual tasks. The past and present studies demonstrate that such a solution could substantially improve the performance and decrease the workload, while the prosthesis operation is perceived as intuitive by the subjects.

## Conclusions

The BPC is one of the first systems that addressed the bimanual ADLs, which were until now largely neglected in literature. A prosthesis that actively supports bimanual activities could motivate the users to utilize the prosthesis in a larger set of tasks, thereby increasing the overall utility of the device and decreasing the rejection rates that are still very high [27, 28].

During ADLs, the majority of manipulation tasks require collaboration between two hands in which both limbs should be coordinated in space and time [22, 29]. However, these tasks have been seldom taken into consideration for building more intuitive human-machine interfaces for amputees. Other researchers have approached the problem of bimanual manipulation by using machine learning techniques that allows a larger adaptability to different tasks [21, 30–32]. The results are promising since they may provide a method that could adapt to the large temporal and spatial variability found in bimanual manipulation. However, these methods are yet to be robust and reliable for daily usage of amputees. In the approach presented here, we were able to integrate a simple, yet useful, method that can be easily used by the amputee to improve their quality of life.

## Data Availability

All data are available upon request to the corresponding author, at the address: marko.markovic@med-uni.goettingen.de. Alternatively, the data can be also downloaded directly under the following address: StudyData.

## Abbreviations

BI-ASYNC: Bimanual Asynchronous
BI-SYNC: Bimanual Synchronous
BPC: Bimanual prosthesis control
DoF: Degrees of Freedom
EMG: Electromyography
IMU: Inertial Measurement Unit
MVC: Maximal Voluntary Contraction
SoA: State-of-the-Art
TLX: NASA Task Load Index
UNI: Unilateral
